# A Stress and Pain Self-management mHealth App for Adult Outpatients With Sickle Cell Disease: Protocol for a Randomized Controlled Study

**DOI:** 10.2196/33818

**Published:** 2022-07-29

**Authors:** Miriam O Ezenwa, Yingwei Yao, Molly W Mandernach, David A Fedele, Robert J Lucero, Inge Corless, Brenda W Dyal, Mary H Belkin, Abhinav Rohatgi, Diana J Wilkie

**Affiliations:** 1 Department of Biobehavioral Nursing Science University of Florida College of Nursing Gainesville, FL United States; 2 Division of Hematology and Oncology Department of Medicine University of Florida Gainesville, FL United States; 3 Department of Clinical and Health Psychology College of Public Health and Health Professions University of Florida Gainesville, FL United States; 4 Diversity, Equity, and Inclusion UCLA School of Nursing Los Angeles, CA United States; 5 Department of Family, Community, and Health System Science University of Florida College of Nursing Gainesville, FL United States; 6 School of Nursing MGH Institute of Health Profressions Boston, MA United States; 7 College of Medicine University of Florida-Jacksonville Jacksonville, FL United States

**Keywords:** sickle cell disease, self-management, stress, pain, opioid use, analgesics, intervention, support, protocol, randomized controlled trial

## Abstract

**Background:**

This paper describes the research protocol for a randomized controlled trial of a self-management intervention for adults diagnosed with sickle cell disease (SCD). People living with SCD experience lifelong recurrent episodes of acute and chronic pain, which are exacerbated by stress.

**Objective:**

This study aims to decrease stress and improve SCD pain control with reduced opioid use through an intervention with self-management relaxation exercises, named You Cope, We Support (YCWS). Building on our previous findings from formative studies, this study is designed to test the efficacy of YCWS on stress intensity, pain intensity, and opioid use in adults with SCD.

**Methods:**

A randomized controlled trial of the short-term (8 weeks) and long-term (6 months) effects of YCWS on stress, pain, and opioid use will be conducted with 170 adults with SCD. Patients will be randomized based on 1:1 ratio (stratified on pain intensity [≤5 or >5]) to be either in the experimental (self-monitoring of outcomes, alerts or reminders, and use of YCWS [relaxation and distraction exercises and support]) or control (self-monitoring of outcomes and alerts or reminders) group. Patients will be asked to report outcomes daily. During weeks 1 to 8, patients in both groups will receive system-generated alerts or reminders via phone call, text, or email to facilitate data entry (both groups) and intervention use support (experimental). If the participant does not enter data after 24 hours, the study support staff will contact them for data entry troubleshooting (both groups) and YCWS use (experimental). We will time stamp and track patients’ web-based activities to understand the study context and conduct exit interviews on the acceptability of system-generated and staff support. This study was approved by our institutional review board.

**Results:**

This study was funded by the National Institute of Nursing Research of the National Institutes of Health in 2020. The study began in March 2021 and will be completed in June 2025. As of April 2022, we have enrolled 45.9% (78/170) of patients. We will analyze the data using mixed effects regression models (short term and long term) to account for the repeated measurements over time and use machine learning to construct and evaluate prediction models. Owing to the COVID-19 pandemic, the study was modified to allow for mail-in consent process, internet-based consent process via email or Zoom videoconference, devices delivered by FedEx, and training via Zoom videoconference.

**Conclusions:**

We expect the intervention group to report reductions in pain intensity (primary outcome; 0-10 scale) and in stress intensity (0-10 scale) and opioid use (Wisepill event medication monitoring system), which are secondary outcomes. Our study will contribute to advancing the use of nonopioid therapy such as guided relaxation and distraction techniques for managing SCD pain.

**Trial Registration:**

ClinicalTrials.gov NCT04484272; https://clinicaltrials.gov/ct2/show/NCT04484272

**International Registered Report Identifier (IRRID):**

PRR1-10.2196/33818

## Introduction

### Background

Sickle cell disease (SCD) is an orphan disease with multifactorial impact. In the United States, the incident rate of SCD has remained constant [1]. However, SCD is projected to increase from 304,800 newborns annually to 404,200 newborns by 2050 worldwide [2]. SCD is the most common inherited blood disorder in the United States [3], with annual health care cost of US $2.4 billion [4] for 100,000 diagnosed Americans. Current human diasporas and massive migration from Africa, Middle East, Mediterranean region, Central and South America, and India contribute to the sickle cell gene pool in the United States, as does a growing Hispanic population [2]. People living with SCD were once destined for recurrent morbidity or early death, but now, live into their forties and beyond [5]. In the United States, for people with HbSS, HbSβ^0^, or HbSD, the median age of survival is 48 (95% CI 44.4-58.4) years and for people with HbSC or HbSβ^+^, the median age of survival is 54.7 (95% CI 38.6-62.9) years [6]. People living with SCD have lifelong recurrent episodes of acute pain and substantial persistent pain together with other symptoms including respiratory threats, organ failure, stroke risk, and stress and disruption in their lives and those of their family [7-15]. Evidence indicates that patients with SCD who survive beyond childhood continue to live most days with persistent pain that can affect any organ owing to ischemia [13].

The chronic pain associated with SCD is often rated as more intense than childbirth pain and persists for years [16]. Recurrent, unpredictable, and disabling episodes of vaso-occlusive pain was the primary reason for >100,000 annual SCD emergency department (ED) encounters and hospitalizations [17]. People with SCD have an average of 2.59 (95% CI 2.53-2.65) ED visits or hospitalizations per year [17]. ED visit and hospitalization rates are 2 to 6 and 7 to 30 times higher, respectively, among African American individuals with SCD visits than those without SCD [18].

Other studies report strong positive relationship between the frequency and severity of pain and death [13,19-22]. Currently, the only cure for SCD is allogeneic hematopoietic stem cell transplantation, with limited availability owing to the risk of transplantation-related mortality [23]. Potential cures for SCD include transferring the causal β-globin gene, editing sickle β-globin mutation, or reactivating fetal hemoglobin, the research for which is still ongoing [24]. As stem cell transplantation is not without considerable risk, it is usually reserved for patients with severe symptoms of SCD [25]. Given the strong association between severe SCD pain and death [13], there is an urgent need to find readily available therapies for controlling SCD pain.

Stress, associated with pain in patients with SCD, is correlated with overactivation of the hypothalamic-pituitary-adrenal axis hormones or neurotransmitters (eg, cortisol, norepinephrine, and epinephrine), which trigger the fight-or-flight responses and intensify responses to nociceptive pain (somatic and visceral tissue damage) [26-28]. Stress can also activate neuropathic pain mechanisms (neural tissue damage) implicated as part of SCD pain [16]. In patients with pain, the perception of stress can lead to stress-induced hyperalgesia (increased pain from a painful stimulus) [29]. Findings from studies of patients with SCD [30,31] and other pain conditions [32] show small to medium positive associations between stress and pain. In a pilot study of 52 adult patients with SCD, we found that compared with patients who perceived low psychological stressors from their physicians, those who perceived high stressors from their physicians also reported greater stress (*P*<.001) and pain (*P*=.002) [30]. Results were similar for psychological stressors from nurses (perceived stress: *P*<.001 and pain: *P*=.02) [30]. Others found that patients with SCD who reported high stress also reported high average pain [31], and increased stress intensity was associated with increased same-day pain in patients with SCD [33]. Cumulative findings from these studies provide strong evidence of the association of stress with pain in patients with SCD. The hypothalamic-pituitary-adrenal axis theory posits that stress reduction interventions have the potential to reduce SCD pain.

### Nondrug Therapy for Self-management of Pain in SCD

The use of pain self-management in SCD has gained much attention in recent years. Pain self-management techniques can include deep breathing exercises, progressive relaxation, and guided imagery [34]. Patients with SCD are ideally suited for nondrug therapies. In a recent study of 227 patients with SCD, approximately 92% reported using nondrug therapy in the previous 6 months to manage pain [35]. Studies of nondrug therapies in patients with SCD found reduced perceived pain intensity [36] and reduced clinical pain [37,38]. Guided relaxation, which is one of many tools used in cognitive behavioral therapy, is designed to help patients identify triggers for pain, modify problematic emotions or behaviors, and develop coping skills [39]. By using relaxation and stress management to guide patients away from negative feelings and behaviors, we can weaken the inclination to use opioids as the only method of pain management and strengthen their repertoire of strategies with alternative healthy actions, thereby improving their day-to-day relationship with pain.

### Current Drug Therapy for Managing SCD Pain (Opioids)

The use of opioids as the mainstay of therapy for both acute and persistent SCD is not ideal. Opioid analgesics are known to cause severe side effects, including death from overdose. They are frequently prescribed for SCD pain; however, accumulating evidence suggests the need to incorporate nondrug therapies as strategies for managing SCD pain systematically [40]. A recent study characterized opioid analgesic use in patients with SCD and showed that 40% of them were prescribed opioids in the previous 12 months [41]. Furthermore, 3% of pediatric patients and 23% of adult patients used high doses (>30 mg of oral morphine equivalence [OME] daily) [41]. Findings also suggest that patients with vaso‐occlusive pain crisis (acute) and avascular necrosis (chronic) reported using high-dose opioid use [41].

Patients who use high opioid doses were more likely to use nonsteroidal anti‐inflammatory drugs, visit acute health care facilities, and indicate that they experienced pain on most days [42]. The opioid epidemic in the United States stems from heavy reliance on opioid therapy for controlling all pain types, including persistent pain, a practice against the Center for Disease Control recommendation that nondrug therapies are preferable for treating chronic pain [43]. Compared with the national average, patients with SCD have lower opioid addiction rates and fewer deaths from overdose [44]. Between 1993 and 2013, a total of 174,959 individuals in the United States died from opioid prescription overdose. During the same period, 95 patients with SCD died from opioid prescription overdose [44]. To mitigate the risk of death from opioid overdose and other side effects of opioids in patients with SCD, behavior change strategies are sorely needed, which will empower patients to embrace self-management principles, including the systematic use of nondrug therapies as complements to opioids.

Providing behavior change strategies via mobile health (mHealth) apps or internet-delivered interventions aligns with the Federal Pain Research Strategy to promote and enable pain self-management [45]. In a previous study, Ezenwa et al [37] found that an internet-enabled, stand-alone distraction and relaxation intervention demonstrated the feasibility and acceptability of the intervention in adult outpatients with SCD. As in this previous study [37], the design for this study is based on a multidimensional theory of pain supported by decades of research [46]. Operating within this theoretical framework, participants with SCD will continue taking opioid analgesics during the study period. A well-designed study, such as ours, will provide a behavior strategy with the potential of reducing opioid use.

### Study Aims and Hypotheses

The focus of this study is to evaluate the effectiveness of a self-management intervention that promotes the use of relaxation and distraction exercises (RDEs) in reducing pain, stress, and opioid dependency among adult outpatients with SCD. Specifically, the aims of this study are the following:

1. Determine the *short-term* effects of You Cope, We Support (YCWS). Hypothesis: In the first 8 weeks, compared with the control group, the experimental group will report reductions in the primary outcome—pain intensity (0-10 scale) and in the secondary outcomes—stress intensity (0-10 scale) and opioid use (OME).
2. Determine the *long-term* effects of YCWS. Hypothesis: During months 3 to 6, compared with the control group, the experimental group will report reductions in the primary outcome—pain intensity (0-10 scale) and in the secondary outcomes—stress intensity (0-10 scale) and opioid use (OME).
3. Use machine learning to develop and evaluate models that predict patient outcomes based on their group assignment and their personal (eg, self-efficacy, sex, education, family income, and computer experience) and environmental characteristics (eg, distance from care and quality of internet connection). The results will provide insight into the heterogeneity of intervention’s effects and the patients most likely to benefit from YCWS.

## Methods

### Study Design

The study is a randomized controlled trial (RCT) of the short-term (8 weeks) and long-term (6 months) effects of YCWS on efficacy outcomes (pain, stress, and opioid use), designed to determine the short-term and long-term effects of the YCWS intervention and to evaluate models that predict patient outcomes based on their group assignment and their personal and environmental characteristics.

### Study Setting

We are recruiting patients both in person and internet-based throughout the state of Florida, including the University of Florida (UF) Health-Shands in Gainesville and UF Health-Shands in Jacksonville. Thus, data collection and intervention delivery occur throughout Florida, with UF Health-Shands in Gainesville as the primary site for data collection, intervention delivery, and data analysis.

### Ethics Approval

The UF institutional review board (IRB) is the approving IRB of record and has approved all the recruitment and study procedures (IRB202000984).

### Recruitment

We will recruit from the UF Health-Gainesville and UF Health-Jacksonville hematology clinics and the UF Health-Jacksonville transition program. The UF Health-Shands Hospital, Gainesville, adult sickle cell program has a clinic panel of 497 adult patients with SCD. Of the 497 patients, (283/497, 57%) are women and (452/497, 91%) are African American individuals. The adult SCD program consists of a multidisciplinary team including 5 medical hematologists, 2 advanced registered nurse practitioners, 1 physician assistant, and 1 registered nurse. The UF Health-Jacksonville adult sickle cell program has a clinic panel of 569 adult patients with SCD. Of the 569 patients, 318 (56%) are women and 540 (95%) are African American individuals. The UF Health-Jacksonville adult SCD program consists of a multidisciplinary team including 1 medical hematologist and 1 advanced registered nurse practitioner.

The study is registered on ClinicalTrials.gov. We will use web-based recruitment processes, including posting recruitment information on the UF study Facebook page and various internet listservs to identify and recruit adults with SCD from across Florida. We will also use colorful posters, flyers, and brochures developed for this study and distribute them in sickle cell clinics and health fairs and through university bulletin boards. In addition, we will work directly with sickle cell organizations, community-based organizations, health care networks, and churches to post recruitment flyers aimed at encouraging adults to participate in and advertise the study within their networks throughout Florida.

### Eligibility Criteria

#### Inclusion Criteria

To be included in the study, patients must be adults with a diagnosis of SCD (eg, HbSS, HbSC, HbSβ^0^ thalassemia, and HbSβ^+^ thalassemia), report moderate to severe level of pain (>3 on 0-10 scale) related to SCD within previous 24 hours, use opioid analgesics on *as-needed* or *continuous* basis, speak and read English, and be ≥18 years.

#### Exclusion Criteria

Patients will be excluded if they are legally blind or physically unable to complete the procedures or have previously participated in our relaxation and distraction intervention feasibility study.

### Sample Size

For those who meet the eligibility criteria, we anticipate enrolling 195 participants to retain 170 (87.2%) participants with complete data at 8 weeks, based on a 13% attrition rate observed in our previous longitudinal studies in which follow-up was more than a year. We will strive for a sample in which 95% participants have African descent and approximately 50% are women. Our team has been exceptionally successful in conducting longitudinal studies using tablet technology with this vulnerable population and recruiting and retaining patients with SCD for pain research [16,47-50].

### Screening and Consent

This is an internet-based study, with all screening and consent processes completed by the research specialist (RS) or the community health worker either in person, internet-based, through email, or through US postal service, depending on the patient’s needs. The RS or community health worker will explain the study to the patients and ascertain their willingness to participate. Patients will be informed that their participation is voluntary and that a decision to decline participation will not affect their care. Patients who choose to participate will be asked to sign a written consent form. We will use one of the following methods: (1) patient will sign the consent form in person and will be given a copy, which they may keep for their record; (2) we will email the consent form for the patient to sign and email back to the RS; or (3) we will send 2 copies of the consent form with a self-addressed envelope with paid postage to the patient using the address provided. The patient will sign the consent form, keep a copy for their record, and mail a copy back to us. Patients recruited via phone or email will sign the written consent form on the day of their first study visit, if occurring in person. Otherwise, patients will sign the consent form electronically or via the other consenting methods described previously. The RS who will assist individuals in these screening and consent processes will be trained in good clinical practices for research and IRB and Health Insurance Portability and Accountability Act procedures. The training includes role-playing with the principal investigator (PI) and retraining if the staff deviate from the proper processes.

### Randomization

We will use permuted block randomization with stratification based on worst pain intensity (≤5 or >5) to assign the 195 consecutively selected patients to the 2 study groups. The assignment will be blind until a sealed electronic envelope is opened after the patient completes the pretest data collection procedures.

### Study Retention and Adherence

An important retention strategy is to engage the patients as active partners in the study by explaining the importance of their contribution. We have built trusting relationships with the clinic staff and patient panel, which facilitated the success of our feasibility study and an 18-month longitudinal study (1R01HL124945) that have resulted in several publications [50-55]. Other important retention and adherence strategies include contacting participants for data collection and updating contact information every month. We hired a health care worker from the community to serve as the study support staff for the YCWS intervention. We will send electronic birthday cards and birthday wishes via email and text message. We will also obtain participants’ addresses, telephone numbers, and 3 other secondary contacts to track them during the 6-month study. We will provide incentives to motivate individuals to enroll in the study, maintain interest, and sustain participation for 6 months of self-monitoring or YCWS. All participants will retain the US $150 Galaxy tablet they use for the study as acknowledgment of their time and participation. We will pay US $50 per month as internet access costs for each participant for 6 months. At the end of 6 months, the total incentive is US $450 per participant, which includes the study tablet and accessory cost. In addition, we will use other general study procedures, including (1) text messaging, video messaging, emailing, or calling participants to remind them if they have not provided data for the day and to encourage them to do so and (2) updating patient addresses and phone numbers as needed during each contact.

### Interventions

#### Experimental Arm

The experimental arm includes self-monitoring of pain and stress, electronic monitoring of opioid use, alerts or reminders, and use of YCWS (3 video banks of RDE and support). The YCWS app features self-monitoring, video banks, and support elements, which comprise the YCWS intervention (Tables 1 and 2).

**Figure 1:**
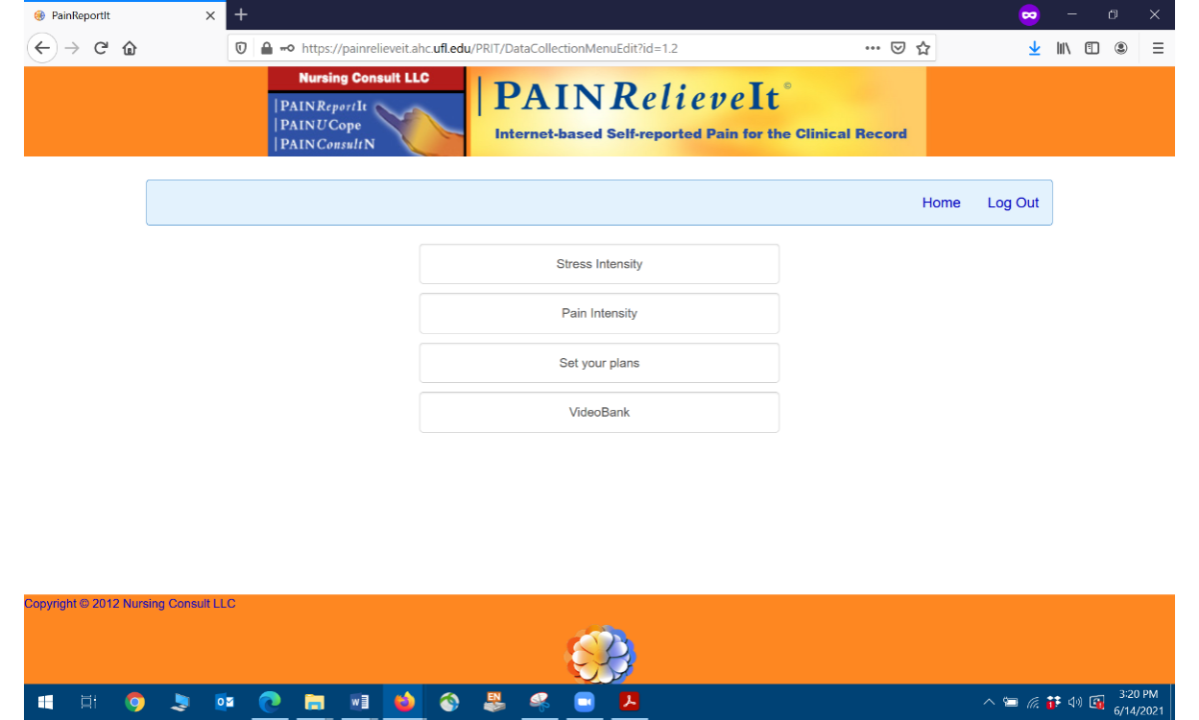
Screenshot of daily stress and pain monitoring for the experimental group.

**Figure 2:**
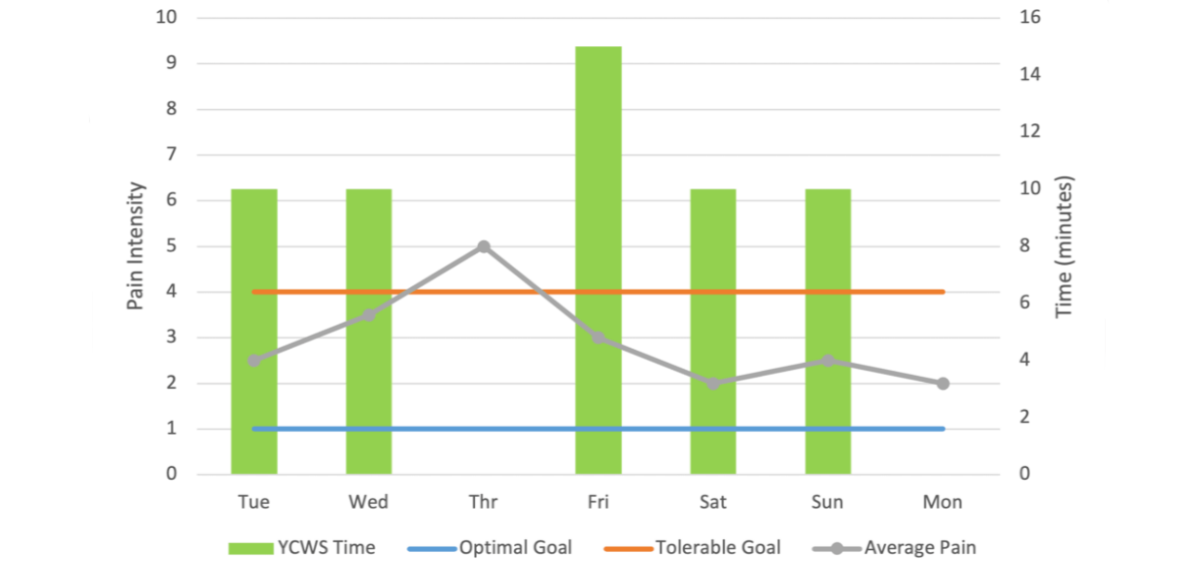
Exemplar Feedback via Graphical Readout for Pain Intensity. YCWS: You Cope, We Support.

When patients log in to the system, they can select the stress intensity scale (0-10) and pain intensity scale (0-10), set plans, or video banks subtabs that display on the screen consecutively (Figure 1). Patients will touch the screen to record their stress and pain intensities. Once their stress or pain intensity is recorded, they will receive instantaneous feedback via graphical readout (Figure 2 presents an approximate model of the graphical readout) showing their stress and pain during the past week (including the present day), pain goals, and daily YCWS use. An auxiliary icon under self-monitoring shows *Set your plans (Goal setting)*. The patient can use this auxiliary icon to revise optimal and tolerable pain goals and time of day to complete study activities including support and to set alerts or reminders. When patients click *video banks* (Table 3), 3 subicons (basic, everyday, and favorite) will be displayed.

**Table 1:** Proposed links between YCWS^a^ app features, examples of YCWS self-management intervention, and self-management skills.

YCWS module	YCWS app features	Examples of YCWS intervention self-management activities	Self-management skills
1	View or update and self-monitor goals, profile, and activities	Patient self-monitors, that is, sets goals for time of day for daily tracking of stress, pain, and opioids use; uses YCWS intervention; or searches for other video links for everyday videos. Patient sets pain control goals regarding optimal pain goal and tolerable pain goal. Patient sets time of day to receive system-generated alerts and reminders, if they have not completed data entry for the day. Patient records self-monitoring data for stress, pain, and opioids use. Patient records searches for other video links for the everyday videos. Patient records time to contact study support staff for troubleshooting issues with YCWS app.	Goal setting and action planning
2	Use video banks for stress and pain reduction	Patient views videos as needed. Patient identifies which of the video links produce stress and pain reduction that met their optimal and tolerable pain goals that were set in module 1 and decides whether to use the same video link multiple times or try another video link.	Action planning and decision-making
3	Get support; patient control panel; receive system-generated alerts or reminders; and contact from study staff, if no data are entered after 24 hours	Patient reviews reminders (eg, how many minutes are left before the next recording of stress, pain, opioids use or to use video banks) or documents questions for study support staff about barriers to and facilitators of self-management. If the patient does not enter data after 24 hours, the study support staff will contact them 2 hours before the next data collection time for troubleshooting. Patient conducts study for the day or responds to support staff about any barriers to completing the study activities as set in self-monitoring goals.	Action planning, problem solving, and decision-making

^a^YCWS: You Cope, We Support.

**Table 2:** Proposed links between YCWS^a^ app features and examples of control self-management activities.

YCWS module	YCWS app features	Examples of control self-management activities
1	View or update and self-monitor	Patient self-monitors by daily tracking of stress and pain. Patient records self-monitoring data for stress and pain.
2	Get support, patient control panel, and receive system-generated alerts or reminders	Patient reviews alerts or reminders (eg, how many minutes are left before the next recording of stress and pain). If the patient does not enter data after 24 hours, the study support staff will contact them 2 hours before the next data collection time for troubleshooting.

^a^YCWS: You Cope, We Support.

**Table 3:** Description of You Cope, We Support banks of video links.

Study period and basic videos			Everyday videos		Favorite videos
**Short term**					
	A total of 6 video clips of guided relaxation and distraction exercises of various lengths: 2, 5, 8, 10, 15, and 20 minutes	Links to investigator-vetted relaxation and distraction exercises of various lengths that are freely available on YouTube		Initially, it will be an empty portfolio. Patients will store their favorite relaxation and distraction video links, which they can choose from basic videos and everyday videos.	
	Used all through week 1 to become familiar with the intervention and learn deep breathing exercise through instructions	Used during weeks 2-8		Used during weeks 2-8.	
	A total of 4 active components of the relaxation and distraction exercises: Soothing female voice Smoke images that change shape Deep breathing exercise Background sound	A total of 4 active components varied across two categories. Category 1: Either male or female voices Variety of nature images Background nature sounds Deep breathing exercise Category 2 Variety of nature images Background nature sounds No voice No deep breathing exercise		On the basis of patients’ preference. Investigators do not control the active components.	
**Long term**					
	Patients will continue to use video bank	Patients will continue to use video bank		Patients will continue to use video bank. Patients will have the ability to find their favorite video clips on the web and add them to favorite videos for personal use and recommend them via the investigators to the community of patients with sickle cell disease. Investigator will sequester and vet links that are not from basic videos and everyday videos to ascertain that they work and are valid before other patients can view them.	

Individuals can click on any of the 3 banks to choose a video to watch. The YCWS intervention can be customized by choosing video clips based on female or male voice, type of nature images, background sound, or lengths and by finding their own video clips. Patients can watch as many video clips as needed. Patients would be able to discern whether they received stress or pain relief after using the videos and determine which of the videos provided them with the most stress or pain relief. According to individual preference, we will send system-generated alerts or reminders to patients or a research staff will call, text, or email the patients to facilitate data entry and intervention use every 24 hours. The patient can also connect with our support staff about issues related to YCWS intervention use (experimental group only) and technical difficulties with study device or app for advice on what to do (both groups). Our support staff will monitor our database daily. If the patient does not enter data after 24 hours of system-generated alerts or reminders or messages via staff cell phone, the study support staff will contact them for data entry troubleshooting and, for the experimental group, for YCWS use as they indicated in their goals. During the short-term trial (weeks 1-8), patients will monitor their stress and pain daily, as described previously.

We will provide experimental group members access to 3 video banks from which they can choose their daily intervention. Patients will use only basic videos during week 1 to become familiar with the intervention and learn deep breathing and relaxation exercises through instructions. For example, one of the basic video clips entitled *Breathing out worry, breathing in light* guides the patients to “Notice the cloud-like formations on the screen. Observe how the images drift and change. As you breathe deeply, let your concerns and tensions (which worry you go) out into the atmosphere where they can dissolve just as the cloud-like formations on the screen dissolve and vanish from view. It is all right to let go of your worries. In fact, it is important to your health that you do so. Just breathe out problems and worries. See them drift off, walking by, and off the screen, no longer a part of you. Now, breathe in sunshine and healing energy.” This requirement is critical and supported by recent evidence showing that the respiratory control center communicates directly with and controls a brain center linked to attention, arousal, and panic [56]. Corless et al [57-60] (Corless, B, unpublished data, January 2022) used these approaches in psychoneuroimmunology studies in patients with cancer or HIV, and we used them in our feasibility studies in patients with SCD. Some of the active components in the basic videos include colorful images similar to smoke or other nature images that slowly change shape against a dark background (images that do not represent any concept with known negative connotations), soothing female or male voice, deep breathing exercise, and slow-paced guided relaxation and distraction instructions. During weeks 2 to 8, patients will also have access to everyday videos and favorite videos.

During weeks 1 to 8, we will send system-generated alerts or reminders or messages every 24 hours to patients in the experimental group via phone call, text, or email to facilitate intervention use. If the patient does not enter data after 24 hours, the study support staff, who will be from the community or RS, will contact them 2 hours before the next data collection time for troubleshooting. The support staff will contact patients via chat, text, email, phone call, or FaceTime based on patients’ preferences to facilitate participation and troubleshoot. Owing to travel burdens including worsening of their pain in cold or hot weather, patient attendance in the monthly SCD support group is extremely low (1-2 patients per month). Patients with SCD report a preference for active remote support as opposed to face-to-face peer or social support because of weather, travel, and parking issues involved with receiving the support at an academic health center. Remote support also empowers them to participate in the study without the additional burden of traveling to the location of any peer or social support as recommended by the Chronic Disease Self-Management Program [61]. We specifically designed the support component of YCWS to incorporate the recommendations from our patient team members to address these issues and refine feedback messages and format.

During the long-term period (months 3-6), only system-generated alerts or reminders via messaging service or staff cell phone will be available. Patients will have the ability to find their favorite video clips on the web, add them to *favorite videos* for personal use, and recommend them via the investigators to other patients with SCD. We will vet these recommendations before releasing them to other patients. Throughout the study period, we will time stamp and track the patients’ YCWS web-based activities to capture data on system use.

#### Control Arm

The control arm includes self-monitoring of pain and stress, electronic monitoring of opioid use, and alerts or reminders. During the efficacy trial (weeks 1-8), similar to patients in the experimental group, patients will monitor their stress, pain, and opioid use daily. We will send system-generated alerts or reminders every 24 hours to patients via phone call, text, or email to facilitate data entry. If the patient does not enter the data after 24 hours of alerts or reminders, the study support staff will contact them for data entry troubleshooting. During the long-term period (months 3-6), patients will continue to self-monitor their daily stress and pain and use the electronic opioid use monitoring device. Patients will also continue to receive system-generated alerts or reminders via messaging service or staff cell phone. Throughout the study period, we will time stamp and track patients’ web-based activities to capture data on system use and outcome variables.

### Protocol Adherence Check for the Fidelity of the Control and YCWS Interventions

The PI will conduct reliability assessments for the RS’s implementation of the protocol on a random sample of 20% of the study measures. Although this assessment cannot be blinded, the RS will not be informed of the reliability check until the baseline data collection procedure is underway. If there are any deviations from the protocol, the RS will undergo additional training and supervised data collection to ensure protocol adherence.

### Intervention Fidelity

To improve fidelity, we will ensure that participants use the YCWS intervention as intended by monitoring the participants’ YCWS system use on the web. During the first 8 weeks, we will call or text participants 2 hours before the next data collection time if the patient does not enter data after 24 hours of system-generated alerts or reminders and has missed the period they indicated for completing the study, to encourage the use of YCWS. We will also ascertain if they are having trouble with accessing the program and will troubleshoot with the participants to resolve any issues. We will also monitor the participants’ self-monitoring of stress, pain, and opioid use in both groups. As a measure of intervention fidelity, the use of the system and measures will be documented by the app software that automatically writes time-stamped data to a SQL database. The fidelity of our pilot feasibility protocol is rigorous as the program is computerized and consistently implemented. In addition, we will monitor mobile device use, and time of use will be documented in the database to determine the intervention dose.

### Outcome Measures

#### Primary Outcome (Pain)

*Pain*, the primary outcome, will be measured using the pain intensity scale. This 3-item scale asks patients to report their current, least, and worst pain intensity in the past 24 hours on a scale of 0 (no pain) to 10 (pain as bad as it could be). We will calculate the average of the 3 scores to obtain an average pain intensity score, which has an internal consistency (*α*) of .85 [62] and predictive validity (Ezenwa, M, unpublished data, April 2022) in the population with SCD.

#### Secondary Outcomes (Stress and Opioid Use)

##### Stress

*Stress* will be measured using the stress intensity scale. This 3-item scale asks patients to report their current, least, and worst stress intensity in the past 24 hours on a scale of 0 (no stress) to 10 (stress as bad as it could be). We will calculate the average of the 3 scores to obtain an average stress intensity score. The internal consistency (*α*) of the stress intensity in the SCD sample was .82 [37]. It showed moderate, but not significant, correlation (*r*=0.41; *P*=.07) with the participants’ Perceived Stress Questionnaire (PSQ)-Recent score, demonstrating fair construct validity [37].

##### Opioid Use

We will use PAINReportIt [63-65] software program (Nursing Consult LLC) to collect data on drug names and doses of scheduled and as-needed opioid analgesics at baseline, and the data will be updated at week 8 and month 6. We will track opioid refills through patients’ pharmacies. We will use the Wisepill medication event monitoring system to collect data on opioid use. The Wisepill records the quantity of the medications. Each patient will receive 2 Wisepill devices, one for scheduled opioids and the other for as-needed opioids. The Wisepill medication event monitoring system was designed to monitor medication adherence and provide instant feedback via cellphone and internet technologies. The Wisepill has been used by patients with various health conditions, including tuberculosis [66], HIV [67], depression [68], and SCD [69]. The dispenser was designed such that it would be compelling and easy to use. It holds approximately 30 large pills or 60 small pills in a 7-compartment inner container and is powered by a 1100 mA lithium polymer rechargeable battery. We will use the Wisepill to record patients’ opioid use. Each week, the patient will fill the Wisepill with their scheduled and as-needed opioid medications on the day of the week indicated in their goals. Subsequently, patients will dispense their daily doses of medication from the Wisepill. Each time the compartment is opened, a cellular signal is sent and recorded in real time on a web-based server. The data are immediately accessible to the research staff for downloading via a secure internet interface. Lucero et al [67] have used this device in previous studies.

#### Descriptive and Process Measures

##### PSQ-Recent

PSQ-Recent [70] is a 30-item tool that measures general perceived stress in the past week. The response options are 1 (almost never) to 4 (usually). Overall perceived stress index score is computed by subtracting 30 from the raw sum score and, then, dividing by 90, yielding scores that range from 0 to 1. High scores indicate great perceived stress. PSQ-Recent has demonstrated good test-retest reliability of 0.82 and good construct validity with the Perceived Stress Scale by Cohen (*r*=0.73) [70]. The internal consistency (*α*) reliability of the PSQ-Recent in our SCD sample was .94 [30]. We will collect these data to understand the context of our intervention.

##### System-Based Daily Activity

We will collect data on patients’ daily activities, such as the time spent on the YCWS intervention, including the time spent in using the video banks of RDE. The software will capture these activities by time-stamped data in the SQL database.

#### Demographic Characteristics

Questions regarding demographic characteristics and analgesics are also included in PAINReportIt [63-65]. Patients will report their age, sex, ethnicity, marital status, level of education, family income, weight, height, physical activity levels (per week), smoking status, stage in the menstrual cycle (women), previous use of computers, current access to computers, distance from care, and quality of internet connection. These data will be collected as composite demographic data to assess the participants’ representativeness and for prediction modeling.

#### Sickle Cell Self-efficacy Scale

The Sickle Cell Self-Efficacy Scale is a 9-item tool that measures patients’ beliefs in their ability to engage in daily activities despite having SCD and coping with obstacles or setbacks associated with SCD. For example, one of the items asks patients, “How sure are you that you can reduce your SCD pain by using methods other than taking extra pain medication?” The response options are 1 (not at all sure) to 4 (very sure). The total self-efficacy score is the sum of the 9 items, with scores ranging from 9 to 36. High scores indicate great self-efficacy. The Sickle Cell Self-Efficacy Scale has been validated in patients with SCD who are ≥11 years and has an internal consistency (*α*) of .87 in the population with SCD [71].

#### Study Acceptability Scale

Patients will also be asked to complete a 6-item questionnaire that focuses on the acceptability of the study [65]. Response options depend on the question. For example, the first question queries “Was participating in this study too hard?” The response choices are (1) not hard at all, (2) somewhat hard, and (3) too hard. This tool has been validated in the population with SCD in a study in which pen tablets were used [65] and in the preliminary study supporting this RCT. This scale provides data on the patients’ thoughts about the study processes and will help plan effectiveness or implementation studies.

#### Exit Interview Guide

This measure contains open-ended questions to solicit patients’ experiences with using a tablet for data collection (3 questions) and opinions (experimental group only) about the YCWS video clips and how they can be improved (3 questions). We will conduct an exit interview about staff support’s acceptability (1 question) and system-generated support (1 question). We will conduct the exit interview at week 8 (RCT-exit; short term) and month 6 (RCT-exit; long term). The exit interview will be audiotaped and transcribed verbatim. We used this measure in our feasibility study. We will use patients’ feedback to modify and improve our protocol for future studies.

### Data Collection and Management

The research data will be collected electronically using laptop and pen tablet computers. All the study surveys may be completed via the web using an app developed for this study. This application will contain PAINReportIt, a computer program developed by DJW to collect participants’ pain information and other surveys in electronic format. Participants’ data will be written directly to that server and will not reside, even temporarily, on the laptop or pen tablet computer. We will use HTTPS for encrypted data transfer to the College of Nursing server. The data will be stored in a secure College of Nursing server with access restricted to the immediate study personnel. All patients will be assigned a code number, and the research team will identify their data only with that code number. The link of the code numbers to the participant identifiers will be kept separate from the study data. Only the investigators and key personnel will have access to the code or master key. Any hard data collected will be stored in a locked office at the College of Nursing.

The software design will make every effort to reduce user errors; all data will be entered directly by the participant using an interface tested with cognitive interview methods that informed the final interface design. Consistency checks are built into the software so that inconsistent data (eg, out of range or logically inconsistent) are flagged immediately to inform the users and assess their intent. In addition, there will be more comprehensive consistency checks before data analysis. Inconsistent data points will be treated as missing. On the basis of our previous studies with this web application, we expect the percentage of inconsistent data values to be very low (<0.5%).

### Statistical Analysis

#### Overview

The study biostatistician will perform data management and analysis in collaboration with the PI and coinvestigators. The data will be stored in an SQL database and exported to the statistical software R for analysis. We will use an intention-to-treat approach, which includes all randomized participants in our analysis. We will consider *P*<.025 as statistically significant for intervention efficacy tests (short term and long term) on the primary outcome of pain intensity. For other inferences, we will consider *P*<.05 to be statistically significant. We will compute descriptive statistics (frequencies, means, SDs, etc) of baseline patient characteristics data, including demographics, pain, stress, and analgesic use before the test and compare the control group and the experimental group using chi-square tests or 2-tailed *t* tests. We expect no significant difference between the 2 randomly generated groups.

#### Aim 1 Analysis

To study the short-term effect of the intervention on the primary outcome (pain intensity) and secondary outcomes (stress intensity and opioid use), all of which are measured daily, we will use linear mixed effects models. Fixed effect terms in the models include the group variable to represent the treatment effect and baseline outcome measurement. Participant-specific random effect terms will be used to account for within-participant correlation in repeat measures. Note that we do not include the time variable in our models because we are primarily interested in the treatment effect on the average pain, stress, and opioid use over time. We do not anticipate significant time trends, but will explore them in the secondary analysis.

#### Aim 2 Analysis

To estimate the long-term effects of the intervention, we will again use linear mixed effects models. Fixed effect terms in the models include the group variable to represent the treatment effect and baseline outcome measurement. Participant-specific random effect terms will be used to account for within-participant correlation in repeat measures.

#### Aim 3 Analysis

We will construct machine learning models, in particular, random forest models, to predict outcomes based on patient group assigned, patient’s personal characteristics (eg, self-efficacy, sex, education, family income, and computer experience), and environmental factors (eg, distance from care and quality of internet connection). A random forest model consists of a large ensemble of regression trees and the average prediction of these trees will be output as the prediction of the model. Regression trees can accommodate nonlinearity and complex interaction without manual specification by researchers, but have the disadvantage of noisiness. Random forest retains the advantage of tree, while reducing noise by introducing randomness in the form of training each component tree with a bootstrap sample of the original training data and randomly selecting a subset of predictors as candidate for splitting at each split of the tree, to reduce the correlation between the trees and the variance of average prediction. We will use a random 70/30 partition (stratified by group and baseline pain) to reserve 30% of the patient sample as the test set, with the remaining 70% as the training set. We will use cross validation to optimize the parameters (minimal node size and number of random features selected for each split) for the random forest prediction models. The models, trained and optimized on the training data set, will be assessed using the reserved test data set to obtain an unbiased estimate of its performance when applied to new data. These models will allow us to identify individuals most likely to benefit from the proposed intervention and the modifiable environmental factors to improve the effects of the intervention.

#### Missing Data

Random effect models can accommodate longitudinal data with missing visits. For visits with partial data (item-level missing data), we will use multiple imputation by fully conditional specification to generate multiple completed data sets, on each of which statistical inference will be performed separately and then aggregated using Rubin rule [72]. This allows us to fully use the information provided by the participants.

#### Sample Power

The success of this study lies on its short-term and long-term efficacy on the primary outcome of pain intensity. We conservatively assume an SD of 3 for pain intensity based on values we observed in earlier studies. With a 2-sided type I error of 0.025, the proposed sample of 170 patients provides 80% power to detect a pain reduction of 1.5, below the minimal pain reduction of 2, which is considered as clinically meaningful.

## Results

The project was funded by the National Institutes of Health and National Institute of Nursing Research. Our IRB approved the study on May 14, 2020.

Study recruitment started on March 29, 2021. As of April 2022, we had enrolled 45.9% (78/170) participants in the study. Of the 78 participants, 65 (83%) participants have completed baseline, 53 (68%) have completed the postintervention assessment after 8 weeks, and 28 (36%) have completed the 6-month study.

## Discussion

### Study Significance

As technology advances, the use of mHealth apps is becoming an integral part of daily life, including for self-management of chronic conditions. Web-based interventions and technologies are reported to have high usability and acceptability as a tool to monitor and self-report daily pain [73-75], monitor medication adherence [76-79], increase SCD reproductive knowledge scores [80,81], and reduce current pain and stress levels [37]. To the best of our knowledge, we are the first to use an mHealth intervention with self-management RDEs to reduce stress and improve SCD pain control, with a concomitant reduction in opioid use.

This protocol paper presents the design of the RADIANCE study, an RCT with the long-term goal to reduce stress and improve pain control in patients with SCD with less opioid use. The intervention with self-management RDEs, YCWS, can offer interactive learning that allows sustained or repeated sessions. Although the intervention may have no direct benefit to individual participants, it may provide individuals with previously unrecognized insights about their stress, pain, and opioid use and options for self-management of these symptoms.

### Strengths and Limitations

A strength of this guided relaxation study is its web-based design and implementation that provides patients with the ability to interact with the program at their convenience in real-world settings. By providing the tools to reduce their pain, it may help patients with SCD to feel empowered. Given that the guided relaxation is web-based, the patients may have a sense of control and empowerment. However, the web-based design can also be a limitation if internet connection becomes an issue. We attempt to mitigate this potential effect by making the intervention platform independent and allowing patients to go to the library and use the library computer or use their other smart devices for the study. Furthermore, the findings of this study will be generalized only to the state of Florida and not nationwide.

Although recruiting patients from health care networks and churches is a new strategy for us in Gainesville, Florida, this will likely be an opportunity for access to affected communities (ie, college students and other young adults). We will report on the patients’ YCWS web-based activities, data on system use, and pain and stress intensity. The study has the potential for assisting people to develop the confidence necessary to self-manage stress that could intensify their acute or persistent SCD pain. If demonstrated to be effective, this internet-based and web-based intervention could be made available nationwide and, eventually, worldwide.

### Dissemination Plan

To maximize the dissemination of this study, we will share methodological approaches, data, and results generated from this study by publishing our findings in research journals that are indexed by PubMed and generally accessible to the research community. We will also present the findings and methodologies at national and international research and health care conferences in accordance with the proposed study time line.

## Abbreviations

ED: emergency department
IRB: institutional review board
mHealth: mobile health
OME: oral morphine equivalence
PI: principal investigator
PSQ: Perceived Stress Questionnaire
RCT: randomized controlled trial
RDE: relaxation and distraction exercise
RS: research specialist
SCD: sickle cell disease
UF: University of Florida
YCWS: You Cope, We Support
